# Grand SLAM study protocol: a prospective randomised multicentre study of shortened versus standard duration adjuvant immune checkpoint inhibition for stage IIB-C, III and IV cutaneous melanoma

**DOI:** 10.1186/s12885-026-15924-4

**Published:** 2026-03-27

**Authors:** Patric Ejder, Henrik Jespersen, Micaela Hernberg, Lars Ny, Ana Carneiro, Sander Ellegård, Kalle Mattila, Roger Olofsson Bagge, Georgios Fountoukidis, Yvonne Brandberg, Mario Mandala, Brent O´Carrigan, Bastian Schilling, Ana Aranche, Omid Hamid, Anders Berglund, Hildur Helgadottir, Gustav Ullenhag

**Affiliations:** 1 Department of Oncology, Uppsala University Hospital, Uppsala, Sweden; 2 Department of Immunology, Genetics and Pathology, Uppsala University, Uppsala, Sweden; 3 Department of Oncology, Oslo University Hospital, Oslo, Norway; 4 Department of Oncology, Helsinki University Hospital, Helsinki, Finland; 5 Department of Oncology, Sahlgrenska University Hospital, Gothenburg, Sweden; 6 Department of Oncology, Skåne University Hospital, Lund, Sweden; 7 Department of Oncology, Linköping University Hospital, Linköping, Sweden; 8 Department of Oncology, Turku University Hospital, Turku, Finland; 9 Department of Surgery, Institute of Clinical Sciences, Sahlgrenska Academy, University of Gothenburg, Gothenburg, Sweden; 10 Department of Oncology, Örebro University Hospital, Örebro, Sweden; 11 Department of Oncology-Pathology, Karolinska Institutet, Stockholm, Sweden; 12 University of Perugia, Santa Maria Misericordia Hospital, Perugia, Italy; 13 Department of Oncology, Addenbrooke’s Hospital, Cambridge, UK; 14 Department of Dermatology, Goethe University Frankfurt, Frankfurt, Germany; 15 Department of Oncology, Hospital Clínic Barcelona and IDIBAPS, Barcelona, Spain; 16 Department of Oncology, The Angeles Clinic and Research Institute, A Cedars Sinai Affiliate, Los Angeles, USA; 17 Epistat, Uppsala, Sweden; 18 Department of Oncology and Pathology, Karolinska Institutet and Theme Cancer, Karolinska University Hospital, Stockholm, Sweden

**Keywords:** Cutaneous melanoma, Immunotherapy, Adjuvant, Neoadjuvant

## Abstract

**Background:**

Adjuvant treatment with PD-1 inhibitors for 12 months has been the established standard of care for patients with resected stage IIB-IV cutaneous melanoma. In other solid tumours (e.g. breast and colorectal), a shorter duration of adjuvant chemotherapy has been shown to be non-inferior with improved toxicity profile. More recently, neoadjuvant immunotherapy with immune checkpoint inhibitors for clinically detectable stage III and stage IV disease has been introduced. There is no clear biological rationale for the chosen duration, and no studies have investigated duration of adjuvant treatment with immune checkpoint inhibitors. A reduced duration of adjuvant therapy could lead to less toxicity from reduced drug exposure, patients returning to normal life sooner, significantly lower drug costs and better healthcare resource utilization. There remains significant interest from patients and clinicians to address this important question.

**Methods:**

Grand SLAM is a prospective phase III randomised, controlled international multi-centre non-inferiority study. The primary objective is to investigate if short (6 months) has equal efficacy as long (12 months) duration of (neo-)adjuvant immune checkpoint inhibition in relation to distant metastasis-free survival and relapse-free survival at landmark analysis at 2 years. After radical surgery of stage IIB-C, III or IV cutaneous melanoma, patients are randomly assigned 1:1 to short or long adjuvant treatment with either nivolumab or pembrolizumab. Patients who have received neoadjuvant treatment with major pathological response are excluded. The sample size of 1,880 patients was determined based on a non-inferiority margin of 4%, a significance level of 0.045 and 80% statistical power. An interim analysis will be conducted when 2/3 of patients are accrued. Biomarkers and the role of food supplements for relapse (MelKo) will be investigated in prespecified substudies.

**Discussion:**

This is the first randomised study to assess a shorter duration of adjuvant anti PD-1 antibody in cutaneous melanoma patients. As of March 2026, the study is recruiting patients in the Nordic countries. Centres in other countries will open shortly.

**Trial registration:**

NCT06488482. Date of registration: 2024-06-10.

**Supplementary Information:**

The online version contains supplementary material available at 10.1186/s12885-026-15924-4.

## Background

The incidence of cutaneous melanoma is rapidly increasing on a global scale, with particularly steep rises in the Western world [1], potentially leading to an increase in the number of patients who will be eligible for systemic perioperative treatment. Since 2018, adjuvant treatment with PD-1 inhibitors for 12 months is routine for stage III melanoma patients, and was based on two pivotal phase III randomised studies, CheckMate 238 and Keynote 054, where the former also included stage IV patients [2, 3]. In 2023, results from two large, randomised phase III studies assessing 12-month treatment with PD-1 inhibitor in patients operated for thick melanoma without lymph node involvement (stage IIB/C) were presented, and the results showed that treatment with PD-1 inhibitors significantly prolonged relapse free survival (RFS) compared to placebo [4, 5], resulting in the implementation of adjuvant treatment in these patient groups in several countries. However, in any of these studies there is no clear rationale for the chosen 12-month adjuvant treatment duration. In addition, results reported from a German study indicates that 6 months of adjuvant PD-1 inhibition is as effective as treatment for 12 months [6].

With PD-1 inhibitor monotherapy, in the neoadjuvant setting, almost 50% of the patients experience a complete or near complete pathological response, while this is the case for more than 60% with combination immunotherapy [7, 8]. A randomised phase II study (SWOG S1801) involving patients with resectable macroscopic stage III and IV melanoma demonstrated significantly improved event-free survival (EFS) in those treated with neoadjuvant PD-1 inhibitors compared to only standard adjuvant therapy. In the experimental arm, patients received three doses of preoperative pembrolizumab 3-weekly followed by up to ten months of the same treatment postoperatively [8]. The phase III study NADINA confirmed the increased benefit of neoadjuvant immune checkpoint inhibition over 12-month adjuvant treatment with PD-1 inhibitor. In this trial, adjuvant treatment was not given to patients where a complete or near-complete pathological response (major pathological response, MPR) following neoadjuvant treatment had been achieved. Other differences compared to the phase II study are that the patients in NADINA received combination immunotherapy (ipilimumab plus nivolumab) neoadjuvant, and that patients with no MPR and a BRAFV600/K-mutated tumour received BRAF- and MEK inhibitors adjuvant [7]. Also in the neoadjuvant studies, the systemic immune checkpoint inhibition was given for 12 months without a clear rationale.

There are also ongoing studies assessing new drugs in the adjuvant setting for high-risk melanoma patients; one phase III study (NCT 05608291) investigates the potential benefit of adding the LAG-3 inhibitor fianlimab to anti PD-1 treatment [9]. In another potential registration study (NCT 05933577), patients who have undergone surgery for high-risk melanoma receive pembrolizumab combined with multiple injections with a neoantigen mRNA-based vaccine [10]. Promising results with this personalized vaccine candidate were shown in a randomised phase II study [11]. Noteworthy, all these trials use a 12-month treatment period.

The current adjuvant treatment practice in melanoma patients varies between countries. In some countries, adjuvant treatment for patients with stage IIB and IIIA melanoma is not recommended, in contrast to the guidelines in several other countries. Whether neoadjuvant treatment has been introduced also varies from country to country [12].

In recent years, adjuvant systemic anti-PD-1 treatment has been introduced for several other cancer types, including non-small cell lung cancer, urothelial cancer, renal cancer and oesophageal cancer. Notably, studies investigating adjuvant treatment durations shorter than 12 months have not been conducted in these patient groups either. The adjuvant systemic treatment in colorectal cancer patients does not consist of immunotherapy but 5-FU based chemotherapy. Interestingly, the treatment duration in these patients has been gradually reduced from two years to three months for oxaliplatin-containing regimens, based on results from large non-inferiority trials [13]. Similar studies have also been conducted for HER2-targeted therapy in breast cancer and their results have enabled a comparable reduction in treatment duration [14]. The rationale for reducing treatment duration may be even more compelling for treatment with immune checkpoint inhibitors (ICI) than for chemotherapy and HER2-targeted therapies. In particular in the neoadjuvant setting, it is common with objective and major pathological responses with only a few doses of ICI [7, 8]. Another difference in opposition to other systemic treatments for tumours is that the ICI therapy can induce memory T cells that might be effective long after treatment stop [15, 16].

A potential major benefit of shorter adjuvant treatment is a decreased risk for severe toxicity, given that such risks accumulate over time. Adjuvant studies have reported grade 3–4 toxicity rates of 10–15% [2, 3]. Additionally, a shorter treatment period requires fewer hospital visits and reduced demand of resources. The cost for checkpoint inhibitors has been estimated to rise from $38 billion in 2021 to $56 billion in 2029 [17]. A large unknown part of this could be referred to adjuvant treatments.

In summary, there is no evidence that a 12-month perioperative systemic treatment regimen is necessary, and the implementation of the current duration was adopted based on registration trials. A key unanswered question is whether a shorter (neo-)adjuvant treatment period is as effective as the 12-month schedule. If reduced duration is as effective, this would clearly benefit patients, significantly lowering drug costs and improve healthcare resource utilization. Therefore, it is highly relevant to conduct a large, randomised study using a non-inferiority design to address this important question.

## Methods

### Study design

**Fig. 1:**
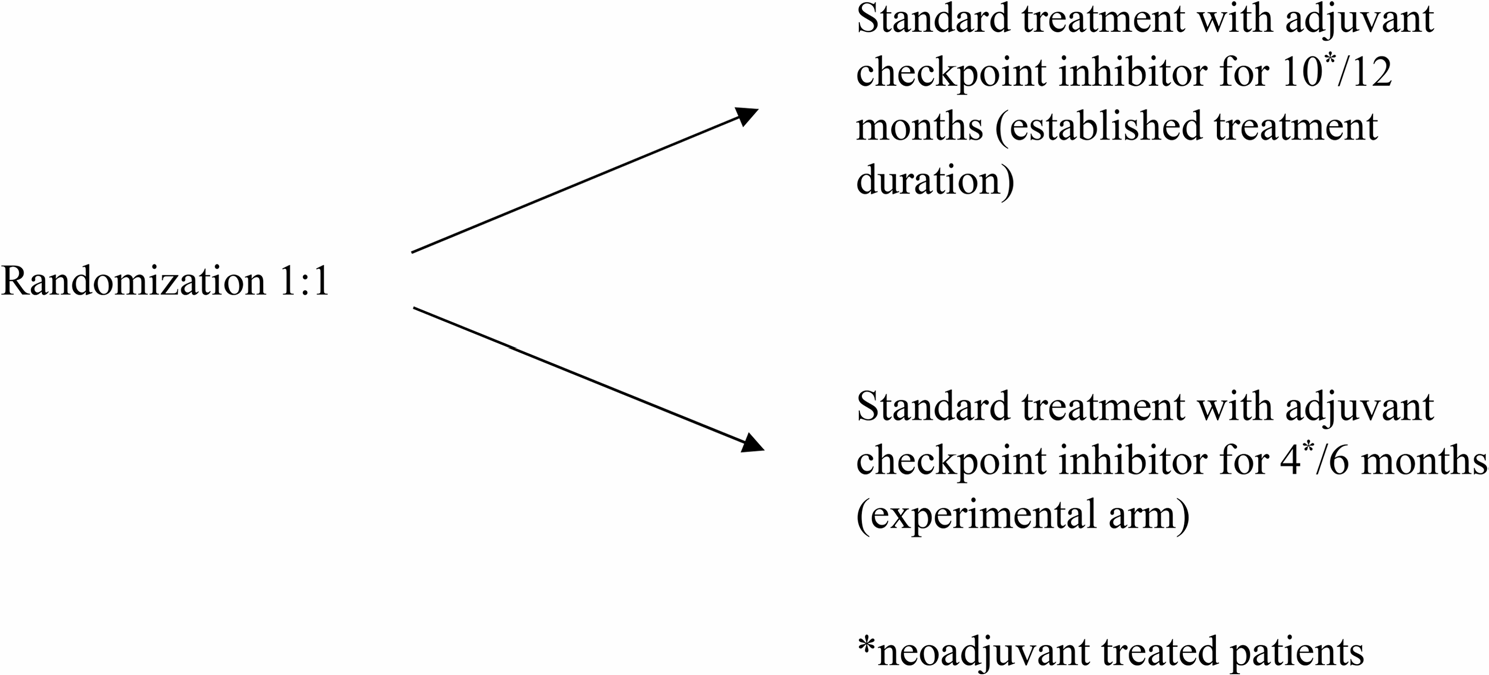
The study design for the Grand SLAM study

The Grand SLAM study is a prospective non-inferior randomised international multicentre study in which patients who have undergone complete surgery for stage IIB/C, III or IV melanoma are randomised to 6 months (experimental arm) or 12 months (standard arm) of (neo-)adjuvant anti-PD-1 inhibitor. All patients will be followed for at least five years. The study design is shown in Fig. 1.

### Primary endpoints

Primary efficacy endpoints are distant metastasis-free survival (DMFS) and RFS at 2 years.

### Secondary endpoints

Secondary efficacy endpoints include long-term DMFS, RFS and overall survival (OS).

### Additional evaluations

Further evaluations will include health economic analyses. In particular, if the experimental arm proves to be as effective as standard treatment, the potential cost savings will be addressed, taking into account not only drug costs but also doctor appointments and nursing time. A detailed analysis plan will be established before data base lock.

### Safety reporting

Since this is a low-intervention trial and patients in the experimental arm most likely will receive a less toxic regimen than the current standard treatment, extensive safety reporting is not considered necessary. Treatment-related serious adverse events (SAEs), occurring under treatment and up to six months thereafter, will be reported. SUSARs will be reported by the sponsor to the authorities based on the information captured in the eCRF.

### Study population

The study population includes patients undergoing complete surgery for cutaneous melanoma (including acral) stage IIB/C, III (including in-transit metastasis) or IV. Patients with stage III melanoma with unknown primary tumour, as well as stage IIB/C melanoma patients who have not undergone sentinel lymph node biopsy (SLNB) procedure, are eligible. Patients who experience a resectable stage III or IV recurrence and have received no adjuvant PD-1 therapy, can also be included. Recruitment is ongoing in Sweden, Norway and Finland and will open shortly in other countries. As of March 2026, twenty-two centres are recruiting patients (see Supplementary File 1).

### Key inclusion criteria

- Written informed consent for participation.
- A complete physical examination within 28 days prior to start of study treatment.
- Performance status ECOG/WHO 0–1 and age over 18 years.
- Adequate organ functions per immunotherapy standard (including sufficient renal function for contrast-enhanced imaging).
- Radical surgery for melanoma (including acral) stage IIB/C, III (including in-transit metastasis) or IV. Patients with stage III melanoma with unknown primary and stage IIB/C who have not undergone SLNB, are also eligible. Patients who experience a recurrence considered surgically resectable (stage III or IV) at a time-point later than the primary diagnosis can also be included.
- Previous adjuvant treatment with BRAF and MEK inhibitors, or perioperative radiation therapy, is allowed.
- Neoadjuvant treatment with ICI up to nine weeks (PD-1 inhibitor +/- ipilimumab) is allowed, unless a complete/nearly complete pathological response was achieved.
- Radiological confirmation of disease-free status within 28 days prior to start of study treatment for patients who have not received neoadjuvant treatment, while 8 weeks is sufficient for neoadjuvant-treated patients.
- Patients must be randomised and start study treatment within 12 weeks after definitive surgery (wide local excision with or without SLNB, lymph-node dissection, or metastasectomy).
- Patients must not use immunosuppressive doses of systemic steroids (> 10 mg/day prednisone or equivalent) for a minimum of 14 days prior to start of study treatment.

### Key exclusion criteria

- Investigator deems the patient unfit for systemic ICI treatment, including a serious or uncontrolled medical condition considered contraindicated by the investigator.
- Active, known, or suspected autoimmune disease. Note: patients with type I diabetes, hypothyroidism requiring only hormone replacement and non-systemically treated skin disorders such as vitiligo, psoriasis, or alopecia, are eligible.
- Other existing or previous malignancy within the past five years, except for in situ breast or cervical cancer treated with curative intent, melanoma, non-melanoma skin cancer and low-risk prostate cancer (the latter also allowed if existing).
- Breast feeding, pregnancy or planned pregnancy.
- Ocular and mucosal melanoma.

### Study procedure and randomization

Randomisation in a 1:1 ratio will be performed locally using an electronic data capture (EDC) system (Viedoc) prior to the start of adjuvant treatment, with stratification based on tumour stage, neoadjuvant treatment given or not, type of neoadjuvant treatment (PD-1 inhibitor +/**-** ipilimumab) and pathological response (partial or non-response) after neoadjuvant treatment.

Adjuvant treatment, with current standard ICI drugs, will be given intravenously or subcutaneously according to routine protocols in both study arms. In addition to registered doses, hybrid dosing is allowed. Specifically, current treatment alternatives include nivolumab, given four-weekly, and pembrolizumab, given three- or six-weekly. Standard treatment may change during the study depending on upcoming results from the previously mentioned ongoing phase III adjuvant studies. Since the neoadjuvant treatment duration is 6–9 weeks, these patients will receive ten months postoperative treatment in the standard arm, or four months postoperative treatment in the experimental arm. In case of treatment delays, the overall duration of treatment will not be prolonged.

### Follow-up

A baseline visit for study information, inclusion and randomisation is scheduled 1–11 weeks after the last melanoma-related surgery. Follow-up visits, blood tests and management of side effects will follow current practice based on national guidelines, typically including physician appointments with physical examination following each radiological assessment. The complete schedule of assessments is provided in Table 1. Imaging is mandated at baseline (within 4 weeks for adjuvant and 8 weeks for neoadjuvant patients respectively, prior to study treatment start), and at 6 and 24 months, where the imaging at baseline and 24 months must include brain imaging. The imaging modality, a CT scan of the thorax, abdomen and brain; CT scan of the thorax and abdomen combined with MRI of the brain; intravenous contrast-enhanced whole body FDG-PET-CT including the brain; or whole body FDG-PET-combined with MRI of the brain, is chosen by the investigator at baseline, with a preference for consistent use. If the primary melanoma was situated on one leg, the FDG-PET-CT must include the site of the primary tumour. In case of a positive sentinel node but where no complete lymph node dissection was performed, CT imaging must include the regional lymph node basin. All patients will be followed for survival for at least 5 years. Extra diagnostic assessments (imaging and laboratory tests) should be performed for any subjects presenting signs and/or symptoms of relapse at the discretion of the investigator and as per clinical practice. Patients in both groups should be instructed to contact their study centre if they experience any symptoms suspicious of recurrence.

**Table 1:** Schedule of assessments

Assessments:	Baseline (a)	3 months (g)	6 months (g)	12 months (g)	18 months (g)	24 months (g)	30 months (g)	36 months (g)	48 months (g)	60 months (g)
Informed consent (b)	X									
Inclusion/exclusion criteria	X									
Demographics	X									
Cancer/treatment history (c)	X									
Concomitant medication	X		X	X	X					
Physical examination (d)	X		X	X	X	X	X	X		
Radiology (e)	X (f)		X			X				
Overall survival follow-up									X	X
SAE		X (h)	X (h)	X (h)	X (h)					
Substudy MelKo			X							
Biomarker substudy (i)	X	X	X	X		X	X			
Pregnancy test (j)	X	X	X (k)	X (k)						

a) Within 28 days of study treatment start. Adjuvant treatment is started within 12 weeks after last melanoma related surgery. Final surgery is defined as excision and/or sentinel node biopsy, lymph-node dissection or metastasectomy

b) Written informed consent must be obtained before any study-specific screening procedures are performed

c) Includes thickness of primary tumour, number of examined nodes/nodes with metastases, TNM staging, postoperative treatment (i.e. adjuvant radiotherapy)

d) To be performed according to clinical routines and this is the follow-up schedule according to current Swedish guidelines for stage III disease

e) CT scan thorax-abdomen-brain/MRI brain *or* i.v. contrast enhanced whole body FDG-PET-CT including brain *or* whole body FDG-PET-CT + MRI brain. Brain must be included in the baseline scan and at 24 months. To be performed according to clinical routines and this is the mandatory minimum schedule

f) To be performed within 28 days for adjuvant and 8 weeks for neoadjuvant patients respectively prior to study treatment start

g) +/- 28 days. Time points from study treatment start, +/- 8 weeks allowed for neoadjuvant treated patients

h) Treatment related serious adverse events during treatment and up to 6 months after treatment will be reported

i) And at relapse

j) Highly sensitive urine or serum/plasma pregnancy test for WOCBP

k) Only applicable for standard arm

### Treatment in the case of recurrence

If recurrence is suspected or verified, the patient should be investigated and treated as per local practice and national guidelines. A discussion at a multidisciplinary team conference (MDT) is encouraged to decide and offer the best available treatment, for example salvage surgery, checkpoint inhibitors, BRAF and MEK inhibitors, radiotherapy, experimental study treatments, or palliative care.

### Statistical methods

Efficacy analyses for DMFS and RFS will be conducted on all per-protocol patients (intention-to-treat population for sensitivity analysis). Distant metastasis free survival is defined as the time from treatment initiation to distant relapse or death, and RFS as the time from treatment initiation to any type of relapse (local, regional or distant) or death. Kaplan-Meier curves will be generated for both endpoints. Comparisons between study arms will be based on a Cox regression model, which will estimate the hazard ratio (HR) and its 95% confidence interval (CI). A detailed description of the Cox regression model will be specified in the Statistical Analysis Plan (SAP). The p-value for testing the null hypothesis, that the HR between the interventions will be greater than or equal to 1.19, will be derived from this model by comparing the log-likelihood of the fitted model with the log-likelihood of a model where the HR between the groups is set to 1.19 by the use of a likelihood ratio test.

### Sample size calculations

The study is designed as a non-inferiority trial investigating whether short-term is inferior to long-term (standard of care) postoperative treatment with ICI. A non-inferiority margin of 4.0% has been established as clinically acceptable for this study population.

The sample size calculation is based on several key assumptions. In the KEYNOTE-716 study, the 2-year DMFS rate for patients with stage IIB/C melanoma was 88.1% [5]. In comparison, the CheckMate 238 trial reported a 2-year DMFS of 70% for patients with stage III–IV melanoma [3]. For the purposes of this calculation, a conservative estimate of 75.0% DMFS at two years was used. The study is powered at 80%, with a significance level (α) of 5% and a non-inferiority margin of 4%. The accrual period is set at 48 months, with an additional 60 months of follow-up.

Assuming a 48-month accrual period and a 60-month follow-up period, a sample size of 1872 patients is estimated to have 80% power, with a one-sided 95% confidence level, to demonstrate non-inferiority with a margin of 4.0% (i.e., between 75% and 71.0% in the observation group). This calculation is based on 1:1 randomization with a corresponding HR of 1.19. The sample size may be re-estimated or the non-inferiority boundary adjusted if concerns arise regarding patient loss to follow-up.

### Co-primary endpoints

Distant metastasis-free survival and RFS are co-primary endpoints. To preserve the overall type I error rate, comparisons will be conducted using a hierarchical testing procedure as follows:

1. The alternative non-inferiority hypothesis, H11: short-term treatment is not inferior to long-term treatment (standard of care) with respect to the first co-primary endpoint (DMFS). If H11 is accepted (i.e., non-inferiority is established for DMFS), then the following hypothesis will be tested:
2. The alternative non-inferiority hypothesis, H12: short-term treatment is not inferior to long-term treatment (standard of care) for the second co-primary endpoint (RFS).

Each test will be performed at a 5% significance level.

### Interim analysis

By allocating a significance level of 0.005 for the interim analysis and 0.045 for the final analysis, the overall sample size increases to 1880 subjects (940 per arm). This calculation was made using the O’Brien-Fleming Boundary. The interim analysis will be conducted when approximately two-thirds of the total number of planned subjects have been enrolled, which represents the optimal timing according to the O’Brien-Fleming approach [18].

### Substudy protocols

Patients included into the Grand SLAM Study may participate in the Grand SLAM Plasma Biomarker Study after providing informed consent. In this substudy we aim to include 100 patients and venous blood samples will be collected at certain time points (Table 1). Plasma and white blood cells will be extracted and immediately stored at − 20 °C and moved to − 80 °C within 2 weeks. For droplet digital PCR and next-generation sequencing (NGS) analysis, cell-free DNA will be extracted from plasma. Chemokine and cytokine profiles will be evaluated, and changes in plasma protein levels as assessed by proximity extension assay will be correlated with circulating tumour DNA (ctDNA) and disease recurrence status. Peripheral blood mononuclear cells will also be analyzed using flow cytometry.

Another side protocol (the MelKo study) to assess the risk of food supplements was added to the TRIM study at oncology centres in January 2021 [19]. The MelKo study only comprises a questionnaire at the 6-month study visit. In March 2026, 313 out of the planned 360 patients had been recruited and MelKo will continue as a substudy to Grand SLAM at Swedish centres.

## Discussion

The incidence of melanoma is rapidly rising worldwide, and the number of patients eligible for perioperative immunotherapy will likely increase. With the current standard adjuvant 12-month treatment for patients with high-risk melanoma, the number needed to treat (NNT) to prevent one relapse is approximately 7, which equals the risk for developing severe adverse reactions (grade 3–5) [2, 3]. Notably, none of the registration studies have to date demonstrated that adjuvant systemic therapy in patients with melanoma significantly improves OS, with its routine use based primarily on prolonged RFS and DMFS [2, 3]. In addition, a recent Swedish cohort study observed no improvement in OS following the introduction of adjuvant ICI treatment [20]. However, a Danish real-world analysis reported a statistically significant OS benefit for patients with stage III melanoma receiving ICI in the postoperative setting compared to matched untreated controls [21]. No prior study has compared a 12-month treatment period with a shorter duration in melanoma patients, making this study the first of its kind.

If the results would demonstrate that a shorter treatment duration is as effective as the current 12-months regimen, this will greatly benefit the patients by potentially reducing the risk for side effects and lower the number of hospital visits. Additionally, the resources saved could be reallocated to other healthcare areas. It is noteworthy that similar studies in breast and colorectal cancer, where adjuvant targeted therapy and chemotherapy significantly prolongs OS, have resulted in shortened adjuvant treatment periods beneficial to both patients and healthcare resource allocation.

The Swedish Melanoma Patient Group (Melanomföreningen) were invited to evaluate the study proposal and supports the study, although they express concerns that patients might be hesitant to participate due to fear of receiving an inferior treatment. While the recruitment rate is expected to be high, given the potential resource savings, such apprehension could nonetheless present an obstacle. Importantly, an interim analysis will be conducted to ensure that the 6-month experimental arm is not clearly inferior to the standard 12-months treatment regimen, in which case the trial recruitment will be halted. However, adjuvant treatment for melanoma is most often given in vain since many patients would not have relapsed even in the absence of adjuvant therapy, and a substantial number of patients relapse despite having received adjuvant treatment. In approximately 20% of patients with metastatic melanoma treated with checkpoint inhibitors a complete response is achieved, and these responses are usually long lasting [22]. This raises the question whether it might be better to refrain from adjuvant immunotherapy and instead reserve treatment for patients who relapse with advanced disease. The patients for whom relapse is prevented by adjuvant PD1-inhibition might be the same patients where a long-lasting complete response is achieved when ICI is given for advanced disease.

The introduction of neoadjuvant treatment with ICI for melanoma is currently underway in many countries, and its introduction is probably only in the beginning. In the Grand SLAM study, this evolving treatment landscape has been taken into account by allowing neoadjuvant treatment within both the 6-month and 12-month treatment arms. If new information emerges that might affect the patients’ informed consent, participants will be re-consented to participating in the study.

In conclusion, there is no evidence that a 12-month perioperative treatment (with or without neoadjuvant therapy) is necessary; the implementation of this treatment length has been primarily driven by registration trials. The important question of whether a shorter adjuvant treatment period, which offers substantial benefits for both patients and health resources, is as effective as the current 12-month schedule has so far not been addressed. Therefore, it is of great interest to perform a large, randomised study with a non-inferiority design, such as Grand SLAM, in order to answer this question.

### Trial status

As of March 2026, the study is recruiting patients in the Nordic countries. Centres in other countries will open shortly and over 70 centres are expected to participate.

## Supplementary Information

Supplementary Material 1.

Supplementary Material 2.

Supplementary Material 3.

## Data Availability

Not applicable yet since the study is ongoing and the results will be presented in the future.

## Abbreviations

OS: Overall survival
DMFS: Distant Metastasis-Free Survival
RFS: Relapse-free survival
GCP: Good Clinical Practice
eCRF: Electronic Case Report Form
CTIS: Clinical Trials Information System.
EDC: Electronic Data Capture
HR: Hazard Ratio
