# Supplementary material for: Grand SLAM study protocol: a prospective randomised multicentre study of shortened versus standard duration adjuvant immune checkpoint inhibition for stage IIB-C, III and IV cutaneous melanoma
Source: BMC Cancer. 2026 Mar 27;26:460. doi: 10.1186/s12885-026-15924-4 (PMC13064366; doi:10.1186/s12885-026-15924-4)
Supplement: Supplementary file 2 — Supplementary Material 2. [file 12885_2026_15924_MOESM2_ESM.pdf]

A prospective randomized international multicenter study to compare Short versus Long Adjuvant immunotherapy after radical surgery of stage IIb-c, III and IV cutaneous malignant Melanoma

SMSG (Swedish Melanoma Study Group), the Nordic Melanoma Group and the EORTC Melanoma Group are in favour of this study.

Running title:

Assessment of short immunotherapy after radical surgery of high-risk malignant melanoma.

Acronym: **Grand SLAM**

Sponsor: Department of Oncology
Uppsala University Hospital
SE-751 85 Uppsala
Sweden

Protocol: Version 5~~4~~.0 2025~~4~~-~~1~~04-07~~8~~

EU CT no: 2023-509994-22-00

ClinicalTrials.gov Identifier: NCT06488482

Principal investigators at the university hospitals of Sweden

Gustav Ullenhag (also coordinating investigator for Sweden and the study overall), Professor, Department of Oncology, Uppsala University Hospital

Hildur Helgadottir (hildur.helgadottir@ki.se), Associate professor, Department of Oncology, Karolinska University Hospital, Stockholm

Ana Carneiro, (Ana.Carneiro@Skane.se), Associate professor, Department of Oncology, Skåne University Hospital

Lars Ny, (Lars.Ny@gu.se), professor, Department of Oncology, Sahlgrenska University Hospital

Ylva Holmgren, (Ylva.Holmgren@regionvasterbotten.se), PhD, Department of Oncology, Norrland University Hospital, Umeå

Georgios Fountoukidis, (georgios.fountoukidis@regionorebrolan.se), MD, Department of Oncology, Örebro University Hospital

Sander Ellegård, (Sander.Ellegard@regionostergotland.se), MD, Department of Oncology, Linköping University Hospital

Investigators at the county hospitals of Sweden

Centralsjukhuset i Karlstad, Emelie Bengtsson (Emelie.Bengtsson@regionvarmland.se), oncologist

Västmanlands sjukhus Västerås, Tania Nicolaescu (Tania-Mihaela.Nicolaescu@Regionvastmanland.se) ~~Cecilia Nilsson (cecilia.nilsson@ltv.se)~~, oncologist

Mälarsjukhuset Eskilstuna, Andreas Nearchaou (Andreas.Nearchaou@RegionSormland.se), oncologist

Sjukhuset i Gävle, Olga val Munos (olga.del.val.munoz@regiongavleborg.se), oncologist

Falu lasarett, Gustav Ullenhag (Gustav.Ullenhag@IGP.uu.se)

Länssjukhuset Sundsvall, Petra Flygare (Petra.Flygare@LVN.se), oncologist

Länssjukhuset Ryhov Jönköping, Mikael Wallander (Mikael.Wallander@Rjl.se) ~~Dimitrios Papantoniou (dimitrios.papantoniou@rjl.se)~~, oncologist

Länssjukhuset i Kalmar, Magnus Lagerlund (Magnus.Lagerlund@ltkalmar.se), oncologist

Länssjukhuset i Växjö, Signe Carlson (signe.carlsson@kronoberg.se), oncologist

Principal investigators at hospitals in Norway

Henrik Jespersen (also coordinating investigator for Norway) (hejes@ous-hf.no), PhD, oncologist, Oslo University Hospital

Cornelia Schuster (cornelia.schuster@helse-bergen.no), PhD, Department of Oncology, Haukeland universitetssjukehus

Jarle Karlsen (jarle.karlsen@stolav.no), professor, Trondheim University Hospital

Anita Amundsen (anita.amundsen@unn.no), North Norway University Hospital, Tromsö

Israr Hussain, (israr.hussain@sus.no), Stavanger University Hospital

Martin Ruppert (Martin.C.F.Ruppert@ahus.no), Akershus universitetssykehus, Ahus

Unn-Miriam Kasti (Unn-Miriam.Kasti@SSHF.no), Sorlandet Hospital, Kristiansand

Torbjørg Standal Skåravik (Torbjorg.Standal.Skaravik@helse-mr.no), Ålesund Sykehus

Principal investigators at university hospitals in Finland

Micaela Hernberg (also coordinating investigator for Finland) (Micaela.Hernberg@hus.fi), Associate Professor, Department of Oncology, Helsinki University Hospital

Veera Sivonen~~Kalle Mattila~~ (Veera.Sivonen~~kalle.mattila~~@varha.fi), PhD, consultant in oncology, Åbo University Hospital

Tanja Skyttä (Tanja Skytta@pirha.fi) ~~Leena Tiainen (Leena.Tiainen@pirha.fi)~~, Tammerfors University Hospital

Okko-Sakari Kääriäinen (Okko.Kaariainen@pshyvinvointialue.fi), Kuopio University Hospital

Other important roles

Statistician: Anders Berglund (anders.berglund@epistat.se), PHD

Radiologist: Eva Penno (Eva.Penno@radiol.uu.se), consultant, Department of Radiology

Senior advisor: Bengt Glimelius (Bengt.Glimelius@IGP.uu.se), professor in oncology, Uppsala

Surgeon: Roger Olofsson Bagge (roger.olofsson@gu.se), professor, Department of Surgery, Sahlgrenska University Hospital

Biomarker study: Kalle Mattila (kalle.mattila@hus.fi) Department of Oncology, Helsinki University Hospital

Correspondence to:

Gustav Ullenhag, Professor

Department of Oncology

Uppsala University Hospital, Sweden

Email: Gustav.Ullenhag@IGP.uu.se

TABLE OF CONTENTS

Table of contents 4

Protocol author and co-ordinating investigator .... 6~~7~~

1 Abbreviations .... 7~~8~~

2 Synopsis .... 8~~9~~

3 Background and rationale .... 9~~10~~

4 Time schedule .... 12~~13~~

5 Aim and hypothesis .... 12~~13~~

6 Evaluation of risk and benefit .... 12~~13~~

7 Recruitment and eligibility .... 13~~14~~

7.1 Recruitment .... 13~~14~~

7.2 Inclusion criteria .... 14~~15~~

7.2.1 Women of childbearing potential (WOCBP) 15

7.3 Exclusion criteria .... 15~~16~~

8 Study procedures .... 16~~17~~

8.1 Randomization procedure .... 16~~17~~

8.2 Study treatment .... 16~~17~~

8.3 concomitant use of other medicinal products and treatments 18

8.4 Study Follow-up Scheme 18

8.5 Treatment in the case of recurrence .... 20~~19~~

8.6 Start, end, temporary halt and early termination 20

8.6.1 Definition of End of Trial 20

9 Evaluations 20

9.1 Primary endpoints 20

9.2 Secondary endpoints .... 21~~20~~

9.3 Additional evaluations .... 21~~20~~

10 Safety reporting 21

10.1 Definitions 21

10.1.2 Serious Adverse Event (SAE) 21

10.1.3 Suspected Unexpected Serious Adverse Reaction (SUSAR) 21

10.2.1 Assessment of causal relationship .... 22~~21~~

10.3 Reporting and registration of Serious Adverse Events (SAE) .... 23~~22~~

10.4 SUSAR 23
11 Statistical methods 23
11.1 Sample size calculations (co-primary endpoint – distant metastases free survival (DMFS) and relapse free survival (RFS)) 24
11.2 Co-primary endpoints 2524
11.3 Interim analysis 25
11.4 HANDLING OF MISSING, UNUSED, AND SPURIOUS DATA AND DEVIATIONS 25
12 Side protocols 26
16.2 Side protocol to investigate the risk of food supplements 26
16.3 Plasma Biomarker Study 26
16.3.1 Rationale for plasma biomarker study 26
16.3.2 Sample collection for ctDNA, proteomics, and immune cell analysis 27
16.4 Other side protocols 27
17 Study requirements 27
18.2 Study organization 27
18.3 Costs and Funding 29
19 Quality control and quality assurance 30
20.2 Monitoring 30
20.3 Source data 30
20.4 Reporting of deviations and serious breach 30
20.5 Adherence to study protocol, ICH-GCP and regulations 31
21 Review by regulatory and ethical authorities 31
22.2 Informed consent 31
22.3 Amendments to study protocol 32
23 Data protection 32
24.2 Data collection, handling and management 33
24.3 Case Report Form 33
25 Insurance 33
26 Study termination and publication 34
27 References 35
28 Appendices 37
APPENDIX 1: Schedule of assessments 38
Appendix 2: DSMB Charter 40

Signature page

Protocol author and co-ordinating investigator

Signature:.. Date:.2024-10-04.............

Gustav Ullenhag, Department of Oncology (YYYY-MM-DD)

Uppsala University Hospital

SE-751 85 Uppsala, Sweden

Protocol approved by the department:

Signature:.. Date:.2024-10-04.............

Henrik Lindman, Head of Department (YYYY-MM-DD)

Section of Oncology, BoT,

Uppsala University Hospital

SE-751 85 Uppsala, Sweden

If you want to join the Grand SLAM study group, please contact project manager Leila Boukharta (leila.boukharta@akademiska.se)

1 ABBREVIATIONS

DMFS	Distant Metastatic Free Survival
RFS	Relapse Free Survival
OS	Overall Survival
CMM	Cutaneous Malignant Melanoma
GCP	Good Clinical Practice
eCRF	electronic Case Report Form
CTIS	Clinical Trials Information System
EDC	Electronic Data Capture
HR	Hazard Ratio
PI	Principal Investigator
WOCBP	Women of child-bearing potential

2 SYNOPSIS

**Clinical Trial Title: A prospective randomized international multicenter study to compare Short versus Long Adjuvant immunotherapy after radical surgery of stage IIb-c, III and IV cutaneous malignant Melanoma**	
**Short Title:**<br>Assessment of short immunotherapy after radical surgery of high-risk malignant melanoma.	
**EU CT number 2023-509994-22-00**	
**Clinical Trial phase**	III
**Clinical Trial Population**	Patients radically operated for stage IIb-c, III and IV cutaneous malignant melanoma (CMM)
**Clinical Trial objective**	To assess whether treatment (adjuvant +/- neoadjuvant) with immunotherapy for 6 months is as effective as 12 months.
**Clinical Trial design**	Randomized non-inferiority
**Study population/indication and total number of subjects (Planned)**	As a screen failure rate of approximately 10 percent is anticipated, screening of approximately 2000 subjects is planned to achieve the target of 1880 subjects in total (940 subjects randomized to treatment in each arm).
**Number of planned clinical trial centers**	Around 630
**Clinical trial duration**	The planned clinical trial duration (from First Subject First Visit to Last Subject Last Visit) is approximately nine years.<br>The planned duration of recruitment (from First Subject First Visit to Last Subject First Visit) is approximately four years.
**Definition of End of Trial**	The trial ends when the last subject has completed the last follow-up (planned Dec 2033).
**Duration of subject participation**	Each subject will be followed for overall survival for at least five years.
**Key Inclusion criteria**	1) Performance status ECOG/WHO 0-1.<br>2) Radical surgery for CMM stage IIb-c, III and IV.
**Key Exclusion criteria**	1) The patient is assessed as unfit to receive immunotherapy.<br>2) An active, known, or suspected autoimmune disease.
**Investigational product**	Standard immunotherapy (adjuvant +/- prestudy neoadjuvant), currently nivolumab or pembrolizumab administered intravenously subcutaneously for 12 months.
**Comparator**	Standard immunotherapy (adjuvant +/- prestudy neoadjuvant), currently nivolumab or pembrolizumab administered intravenously or subcutaneously for 6 months.
**Endpoints**	Primary efficacy endpoints are distant metastatic free survival (DMFS) and relapse free survival (RFS) at 2 years.<br>Secondary efficacy endpoints are long-term DMFS, RFS and overall survival.
**Principal statistical method**	Cox regression model
**Sample size calculation**	1880 patients

3 BACKGROUND AND RATIONALE

The incidence of cutaneous malignant melanoma (CMM) is rapidly increasing in Western Europe, not the least in Sweden. Between 2013 and 2023 the increase was over 60 %, from 3400 to 5500 cases per year in Sweden. According to the Swedish Cancer Society, the number of cases is expected to multiply during the next decades (www.cancerfonden.se). As there are no clear trends of earlier detection of CMM, the number of patients that will be eligible for systemic adjuvant treatment will probably continue to steadily increase.

Since 2017, post-operative treatment with PD-1 inhibitors for 12 months is routine in the Western world for stage III melanoma patients. However, a rational for having chosen a 12-month treatment period in the registration studies is lacking. The introduction of adjuvant immunotherapy for high-risk patients was based on two pivotal phase III randomized studies, CheckMate 238 (nivolumab, Opdivo®) and the other, Keynote 054, (pembrolizumab, Keytruda®) where the former also included stage IV patients (1, 2). In 2023, results from two large, randomized phase III studies assessing immunotherapy in patients operated for thick CMM without lymph node involvement (stage IIb-c) (Keynote 716 and Checkmate 76K) were presented. The results showed that treatment with PD-1 inhibitor significantly prolonged relapse free survival (RFS) compared to placebo (3, 4). Results from neo-adjuvant studies with PD-1 + CTLA-4 inhibitor (OpACIN) have shown promising results but they are small and lack a control arm (5). In addition, results from a randomized phase II study in patients with macroscopic stage III and operable stage IV CMM were published earlier last year showing that the event-free survival was significantly better for patients receiving treatment with neoadjuvant PD-1 inhibitor compared to the standard adjuvant treatment. Patients in the experimental arm received two months of preoperative and ten months of post-operative immunotherapy (6). The ~~ongoing~~ phase III NADINA study ~~aims to~~ confirms the increased benefit of neoadjuvant compared to adjuvant immunotherapy. In this trial, adjuvant treatment was~~i~~s not given to patients where a complete or near complete pathological response on neoadjuvant treatment has been achieved ~~(ClinicalTrials.gov: NCT04949113)~~. Other differences compared to the phase II study is that the patients in NADINA received combination immunotherapy neoadjuvant and that patients with a BRAF-mutated tumor received BRAF and MEK inhibitors adjuvant (7).

Noteworthy, no randomized study has investigated whether a shorter adjuvant treatment is as effective as 12 months. In addition, according to "Clinicaltrials.gov", there is no study addressing this issue.

There are ongoing studies assessing new drugs in the adjuvant setting for high-risk malignant melanoma patients. Two phase III studies investigate Tthe potential benefit of adding thea LAG-3 inhibitor finalimab to anti PD-1 treatment (cemiplimab, Libtayo®) : In RELATIVITY-098, relatlimab is given together with nivolumab (Opdualag®) (NCT05002569) while fianlimab is tested together with cemiplimab (R3767-ONC-2011, Libtayo®) in a phase IIIthe other study (NCT05608291). Furthermore, whether pembrolizumab in combination with the TIGIT-inhibitor, vibostolimab is more effective than pembrolizumab alone is addressed in another large study (KEYVIBE010, NCT05665595). There is yet another potential registration study (INTerpath V940-001, NCT05933577) where patients who have undergone surgery for different stages of high risk CMM, receive pembrolizumab combined with multiple injections with a neoantigen mRNA-based vaccine. Promising results with this personalized vaccine candidate was shown in a randomized (2:1) phase 2 study, KEYNOTE-942 (NCT03897881) (5). All Tthese trials use a 12- month treatment period despite the lack of evidence.

The BRAF and MEK inhibitors dabrafenib and trametinib are alternative adjuvant treatments for patients with BRAF mutated CMM (8). Therapy with the BRAF and MEK inhibitors, encorafenib + binimetinib is being evaluated in another randomized phase III study, COLUMBUS-AD (NCT05270044), in patients who have had radical surgery for thick CMMs without lymph node involvement.

In recent years, adjuvant systemic immunotherapy has been introduced for several other cancer types including non-small cell lung cancer (NSCLC), bladder cancer, renal cancer and esophageal cancer. Interestingly, studies of adjuvant treatment shorter than 12 months have not been conducted in these patient groups either. One plausible reason is that adjuvant studies usually have been conducted with pharmaceutical companies as sponsors.

The adjuvant systemic treatment in colorectal cancer patients is not immunotherapy but 5-FU based chemotherapy. It is noteworthy that in these patients, the length of the adjuvant treatment period has

been reduced gradually from two years to one year to 6 months and presently to 3 months if an oxaliplatin-containing regimen is used based on results from large non-inferiority trials (9). Furthermore, the length of adjuvant treatment with trastuzumab in breast cancer patients has been shortened to 6 months and more recently to 3 months if an oxaliplatin-containing regimen is used based on results from large non-inferiority trials (10).
The current adjuvant treatment practice in CMM patients varies between western countries. In Sweden, adjuvant treatment for stage, IIb, IIc and IIIa patients is not recommended in contrast to the guidelines in several other countries. For example, stage IIc patients are considered for adjuvant treatment in Finland (personal communication with Micaela Hernberg, consultant, Department of Oncology, Helsinki). Whether neoadjuvant treatment has been introduced also varies from county to country. In Sweden, neoadjuvant treatment is recommended according to our national guidelines while it is not recommended in for example Denmark (personal communication with Ingmarie Svane, professor, Department of Oncology, Herlev).

One major advantage with short adjuvant treatment is the decreased risk for severe toxicity since the risk is cumulative. In the adjuvant studies the observed grade 3-4 toxicity is 10-15 % (1, 2). Another patient advantage with a shorter treatment is that fewer hospital visits are needed. One drawback with adjuvant treatment is the resources required, including costs. The cost for checkpoint inhibitors has been estimated to raise from astonishing 24 billion USD in 2021 to 46 billion USD in 2026 (https://www. researchandmarkets.com/reports/5553670/checkpoint-inhibitors-global-market-report-2022#rela0-5446055). A large unknown part of this sum could be referred to adjuvant treatments.

In conclusion, there is still no evidence that a 12-month systemic treatment (adjuvant +/- neoadjuvant) is needed, and the implementation of this treatment length has been done as per registration trials. The important question whether a shorter adjuvant treatment period is as effective as the current one-year schedule has so far not been addressed. A shorter treatment period would clearly be advantageous for patients and lead to a substantial reduction in drug costs and health care resources. It is therefore of great interest to perform a large randomized study with non-inferiority design like the current proposal which is needed to answer the study question.

4 TIME SCHEDULE

2023-2024: Planning, fund-raising, writing the final protocol, setting up the electronic case report form (eCRF) and randomization procedure, gaining ethical committee, radiation protection committee and the Swedish Medical Products Agency approvals.

2023-20254: Recruitment of centers.

2024-2028: Enrollment of patients.

2033: Last patient followed-up for 5 years.

5 AIM AND HYPOTHESIS

The aim is to conduct a prospective non-inferiority randomized international multicenter study with systemic immunotherapy comparing treatment with a duration of 6 months (experimental arm) to 12 months (standard arm) in patients having undergone radical surgery for stage IIb-c, III and IV CMM. The primary aim is to address whether 6 months of immunotherapy (adjuvant +/- neoadjuvant) in these high-risk patients is as effective as the current 12-month scheme. The primary outcome variables are distant metastatic free survival (DMFS) and RFS at 2 years.

The hypothesis is that 6 months of systemic immunotherapy (adjuvant +/- neoadjuvant) is as effective as the current standard treatment scheme of 12 months in patients who undergo radical surgery for high risk CMM.

6 EVALUATION OF RISK AND BENEFIT

With the adjuvant standard treatment today for patients with high-risk CMM, the number needed to treat (NNT) to prevent relapse is roughly seven while adverse reactions grade III-IV develop in around 1/7 patients. This means that the likelihood of benefit equals the risk for developing severe side effects.

If the results show that a shorter treatment period is as effective as the current one-year scheme it would greatly benefit the patients by decreasing the risk for side effects and by reducing the number of hospital visits. In addition, the health care resources saved would enable spending more resources in other areas. The Swedish Melanoma Patient Group (Melanomföreningen) has been addressed and they are in favor of the study, although they anticipate that patients might not want to participate due to fear of receiving an inferior treatment. However, none of the registration studies have so far shown that adjuvant systemic therapy in CMM patients significantly prolongs overall survival (OS)

and its routine use is based on prolonged RFS. As in other malignancies, adjuvant treatment for CMM is most often given in vain as many patients would not relapse even without adjuvant treatment and a large group of patients relapses despite having received adjuvant treatment. Complete response is achieved in around 20 % of metastatic CMM patients and these responses are usually long lasting. This raises the question whether it might be better to refrain from adjuvant immunotherapy and instead save this treatment for those patients who relapse with advanced disease. The patients in whom we prevent relapse by adjuvant immunotherapy may be the same patients who gain from immunotherapy with complete response in the advanced setting. In addition, a recent cohort study did not indicate any OS benefit after the introduction of immunotherapy (11). Importantly, an interim analysis will be conducted to ensure that the experimental arm (6 months of treatment) is not clearly inferior to the standard treatment period of 12 months, in which case the trial recruitment will be halted. Noteworthy, similar studies have been conducted in breast and colorectal cancer patients where adjuvant treatment significantly prolongs OS.

7 RECRUITMENT AND ELIGIBILITY

7.1 RECRUITMENT

Patients will be recruited from treating centers in Sweden and~~ university~~ clinics in other countries, preferably university centers in Europe~~an countries~~. At each participating center, consecutive patients receiving radical surgery for high-risk CMM will be considered for inclusion.
Based on data from the Swedish Melanoma Registry (SweMR), the expected incidence of stage IIIb-d according to AJCC8 classification at the time of diagnosis, is 250 patients/year.
With a recruitment rate of 60%, 150 patients are estimated to be enrolled/year in Sweden only.

In addition, CMM patients diagnosed with stage III at relapse are also candidates for systemic adjuvant treatment. Unfortunately, the annual incidence of patients diagnosed as stage III at relapse is currently not recorded in Sweden. The small number of patients undergoing radical surgery for stage IV CMM is also unknown.
No Swedish stage IIb patients are anticipated to participate since they are not covered by the national recommendation.

We estimate that 50 patients from these subgroups will be included yearly, yielding a total recruitment of 200 patients/year.

7.2 INCLUSION CRITERIA

1) Provision of written informed consent for participation.

2) ≥ 18 years of age.

3) Performance status ECOG/WHO 0-1.

4) Adequate organ functions as per standards for immunotherapy.

5) Radical surgery for CMM (including acral) stage IIb-c, III (including in transit) and IV. Stage III CMM patients with unknown primary and stage IIb-c CMM patients who have not undergone sentinel node procedure are eligible.

6) A complete physical examination within 28 days prior to~~-~~ start.of study treatment~~randomization~~.

7) Previous adjuvant treatment with BRAF + MEK inhibitors is allowed.

8) Neoadjuvant treatment with immunotherapy for two months (currently pembrolizumab every third weeks three times or nivolumab every fourth week two times) is allowed providing that a complete or near complete pathological response was not achieved~~ and patients with clear progressive disease according to the pathology report are not eligible~~.

9) All participants who have not received neo-adjuvant treatment must have disease-free status documented by radiological assessment within 28 days prior to start of study treatment~~randomization~~ ~~-~~while 8~~6~~ weeks is sufficient for neo-adjuvant treated patients.

10) The patient must be randomized and start~~ systemic~~ study treatment within 12 weeks after final s~~S~~urgery (including neoadjuvant patients), i.e. excision + sentinel node biopsy, lymph-node dissection or metastasectomy.

11~~0~~) Participants must be off immunosuppressive doses of systemic steroids (>10 mg/day prednisone or equivalent) for a minimum of 14 days prior to start of study treatment~~drug administration~~.

12~~1~~) Sufficient renal function for radiological assessments with i.v. contrast.

13~~2~~) Peri-operative radiation therapy is allowed.

14~~3~~) Patients who experience a locoregional lymph node relapse, i.e. stage III disease or operable stage IV at a time-point later than primary diagnosis are welcome to participate provided that they have not received neoadjuvant and/or adjuvant immunotherapy.

15~~4~~) Negative highly sensitive urine or serum pregnancy test for women of childbearing potential (WOCBP)

16~~5~~) WOCBP are required to use contraceptives as described below (section 7.2.1)

176) Male subjects should agree to use condom during treatment and for three (3) months after termination of treatment

7.1.1 Women of childbearing potential (WOCBP)

The inclusion of WOCBP (defined as fertile women following menarche and until becoming post-menopausal unless permanently sterile by hysterectomy, bilateral salpingectomy and bilateral oophorectomy), requires use of highly effective contraceptive measures such as:

- Combined hormonal contraception or progesterone-only hormonal contraception associated with inhibition of ovulation (oral, intravaginal or transdermal)
- Intrauterine device
- Intrauterine hormone-releasing system
- Bilateral tubal occlusion
- Vasectomized partner
- Sexual abstinence

Measures for contraception shall be maintained during treatment with IMP, and 5 months after termination of the study treatment.

1.3 Exclusion criteria

1) The patient is, in the opinion of the investigator, assessed as unfit to receive systemic adjuvant treatment.

2) Serious and/or uncontrolled medical disorder that in the opinion of the investigator is contraindicated.

3) An active, known, or suspected autoimmune disease. Participants with type I diabetes mellitus, hypothyroidism requiring hormone replacement only and skin disorders (such as vitiligo, psoriasis, or alopecia) not requiring systemic treatment are eligible.

4) Life-expectancy less than 2 years due to concurrent disease (*e.g.*, cardiac disease and liver cirrhosis).

5) Inability to provide informed consent or refusal to do so.

6) Inability to comply with the study protocol.

7) Participation in other clinical trials interfering with the current study protocol.

8) Existing or previous malignancies within the past 5 years (except for with curative intent treated in situ breast and in situ

cervical cancer, melanoma in situ, malignant melanoma, non-melanoma skin cancer and low risk prostate cancer (the latter also allowed if existing)).

9) Breast-feeding, pregnancy, or planned pregnancy.

10) Ocular and mucosal melanoma.

~~The patient must be randomized and start systemic treatment within 12 weeks after final surgery, i.e. excision + sentinel node biopsy, lymph-node dissection or metastasectomy. However, neoadjuvant treated patients must be randomized within 6 weeks after radical surgery.~~

8 STUDY PROCEDURES

8.1 RANDOMIZATION PROCEDURE

Randomization 1:1 will be locally performed in the electronic data capture (EDC) system (Viedoc) before start of adjuvant treatment and stratified according to tumour stage, ~~and~~ neoadjuvant treatment given or not, type of neoadjuvant treatment (PD-1 inhibitor or ipilimumab + nivolumab) ~~treatment~~ and pathological response (partial or non-response) after neoadjuvant treatment.

8.2 ~~8.2~~ STUDY TREATMENT

Adjuvant treatment with current standard immunotherapy drugs will be given according to the routine treatment schemes in both study arms.

~~Adjuvant +/- neoadjuvant treatment with current standard immunotherapy drugs will be given according to the routine treatment schemes in both study arms. The study treatment options are:~~

- ~~Nivolumab given every second or fourth week at the registered dose, i.e. 240 mg or 480 mg respectively.~~

  ~~Pembrolizumab given every third or sixth week at the registered dose, i.e. 200 mg or 400 mg respectively.~~

  Hybrid dosing is also an alternative which means that patients will receive an individualized dose based on weight according to Table 2 in Malmberg et al (17) and as listed below:

	**Hybrid dosing**
**Pembrolizumab**	100 mg every 3 weeks for individuals weighing <65 kg, or 150 mg for individuals weighing ≥65 kg

	200 mg every 6 weeks for individuals weighing <65 kg; 300 mg every 6 weeks for individuals weighing 65-90 kg; or 400 mg every 6 weeks for individuals weighing ≥90 kg
**Nivolumab**	3 mg/kg every 2 weeks, maximum 240 mg
	6 mg/kg every 4 weeks, maximum 480 mg

The kind of immunotherapy and the dosing interval that is to be used for an individual patient must be decided by the PI before randomization. Subcutaneous route and bisoimilars will be allowed under the prerequsite that they are approved by the EMA.

The standard treatment might change during the course of the study, based on upcoming results from ongoing phase III adjuvant studies (see above). If standard treatment is changed a protocol amendment will be issued and a substantial modification (SM) will be submitted and approved in Clinical trial information system (CTIS) before the new treatment is implemented in the study.

Patients who have undergone neoadjuvant immunotherapy are also eligible for the study providing that a complete or near complete pathological response has not been achieved. ~~which usually is the case~~ With PD-1 inhibitor as single treatment, ~~in~~ almost 52~~2~~0% of the patients experience a complete or near complete pathological response reponse~~cases~~ (6) while this is the case for more than 60% with combination immunotherapy (7). Since the neoadjuvant treatment duration is two months, these patients will in the standard arm receive 10 months post-operative treatment and in the experimental arm, four months post-operative treatment only. ~~In case of treatment delays, the duration of treatment will not be prolonged.~~ Follow-up visits, blood tests and management of side effects will follow current practice based on national guidelines.

Expected side effects of the study treatment (refer to respective Summary if product characteristics, SmPC) will be managed according to routine clinical practice and the international guidelines for the management of immune related side effects: ASCO, ESMO and The society of immunotherapy on cancer (SITC).

Randomization 1:1 →

Standard treatment with checkpoint inhibitor for **10*/12 months** (established treatment duration).

Standard treatment with checkpoint inhibitor for **4*/6 months** (experimental arm).

*neoadjuvant treated patients

Patients who have not received neoadjuvant treatment

In case of treatment delays, the duration of treatment will not be prolonged, regardless the number of treatments administered. The study treatment options are:

- Nivolumab given every fourth week (six treatments in the experimental and thirteen in the standard arm respectively) at the registered dose, i.e. 480 mg respectively.
- Pembrolizumab given every third (eight treatments in the experimental and seventeen in the standard arm respectively) or sixth week (four treatments in the experimental and nine in the standard arm respectively) at the registered dose, i.e. 200 mg or 400 mg respectively.

Patients who have received neoadjuvant treatment

Usually, the stipulated number of treatments will be given on the discretion of the investigator regardless of treatment delays.

- Nivolumab given every fourth week (four treatments in the experimental and eleven in the standard arm respectively) at the registered dose, i.e. 480 mg.
- Pembrolizumab given every third (six treatments in the experimental and fifteen in the standard arm respectively) or sixth week (three treatments in the experimental and seven in the standard arm respectively) at the registered dose, i.e. 200 mg or 400 mg respectively.

1.3 CONCOMITANT USE OF OTHER MEDICINAL PRODUCTS AND TREATMENTS

Medications considered necessary for the safety and well-being of the subject may be prescribed at the discretion of the investigators, unless otherwise specified in the exclusion criteria. Concomitant medication should be recorded in the Case Report Form (CRF).

1.4 STUDY FOLLOW-UP SCHEME

The schedule of assessments is listed in Appendix 1.

A baseline visit 1-11 weeks after the last melanoma-related surgery is scheduled for study information, inclusion and randomization. The national routine schedule for follow-up visits will be

applied for all patients in the study according to standard practice which normally includes doctor appointments with physical examinations after each radiological assessment.

The scheme includes compulsory imaging including brain at baseline (within 4 weeks for adjuvant and 8 weeks for neoadjuvant respectively of study treatment start~~randomization and including brain~~), and at 6 and 24 months ~~(including brain)~~ respectively (see flow-chart below). The minimum imaging scheme is similar to Swedish guidelines but clearly less extensive than current practice in most other countries, e.g. Great Britain (Paul Lorigan, personal communication) and much more frequent imaging was done in previous adjuvant studies (1, 2). Swedish patients will undergo one extra scan (at 24 months) compared to national guidelines which corresponds to (Uppsala University Hospital as reference) an additional radiation dose of 12 mSievert (dose for CT scan) or 6 mSievert (dose for FDG-PET-CT). The mode of imaging (CT scan of the thorax, abdomen and brain/MRI brain or i.v. contrast enhanced whole body FDG-PET-CT including brain or whole body FDG-PET-CT and MRI brain) is chosen by the investigator at baseline and preferably, the same method is used for all imaging. Importantly, if an FDG-PET-CT without contrast or CT not including brain is conducted at 24 months, MRI of the brain must also be performed. If the primary CMM was situated on one leg, the FDG-PET-CT must include the site of the primary tumour. In case of a positive sentinel node but no performed lymph node dissection, CT imaging must include local lymph nodes.

All patients will be followed for survival for at least 5 years.

Extra diagnostic assessments (imaging and laboratory tests) should be done for all subjects presenting signs and/or symptoms of relapse at the discretion of the investigator and as per clinical practice. Patients in both groups should be instructed to contact their study center if they experience any symptoms suspicious of recurrence.

Flow-chart of minimum number of radiological assessments

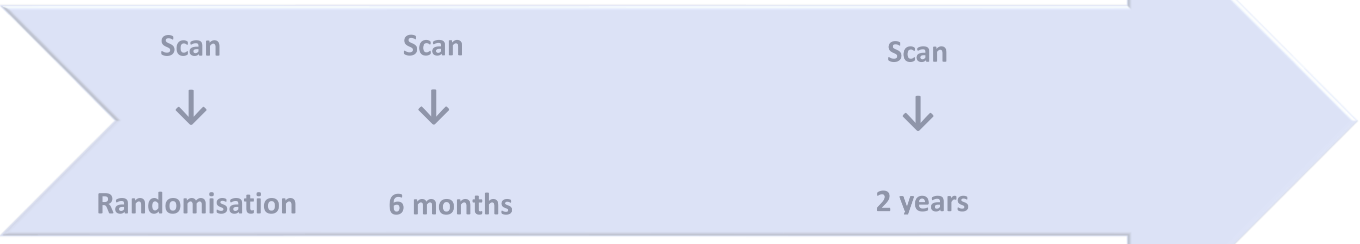

This study will consist of 3 phases: screening, treatment, and follow-up.

1.5 TREATMENT IN THE CASE OF RECURRENCE

If recurrence is suspected or verified, the patient should be investigated and treated as per local practice and national guidelines. A discussion of the case at an MDT conference, in order to decide and offer the best available treatment (e.g. salvage surgery, palliative chemotherapy, checkpoint inhibitors, BRAF- and MEK-inhibitors and/or radiotherapy, experimental treatment in a study, palliative care) is encouraged. The MDT should preferably include a surgeon, a dermatologist, a pathologist, a radiologist, a contact nurse, a research nurse and an oncologist. Patients who experience local recurrence ~~and start a new treatment~~ will continue to be followed up according to the protocol for a total of five years. Patients who experience distant relapse will continue to be followed up for survival according to the protocol for 5 years.

1.6 START, END, TEMPORARY HALT AND EARLY TERMINATION

The start of the trial is defined as the first visit of the first subject. Sponsor will report the start of the trial in each participating country through notification in CTIS within 15 days. The trial may be prematurely terminated for safety reasons affecting the risk-benefit balance or if the recruitment of subjects cannot be completed within reasonable time. Decisions on premature termination are taken by the sponsor. The Competent Authorities will be informed as soon as possible via CTIS, but no later than 15 days after trial suspension.

If the trial is prematurely terminated or suspended, the investigator will immediately inform the subjects and ensure appropriate treatment and follow-up. On the individual level the treatment will be stopped if severe toxicity occurs, please see the international guidelines (ASCO, ESMO and SITC).

1.6.1 Definition of End of Trial

The trial ends when the last subject has completed the last follow-up.

9 EVALUATIONS

9.1 PRIMARY ENDPOINTS

The primary endpoints of the study are DMFS and RFS at 2 years.

9.2 Secondary endpoints

The secondary endpoints are long-term DMFS, RFS and OS. DMFS and RFS are evaluated with radiology and clinically. OS is evaluated based on survival data in patient medical records. For the secondary endpoints, the patients will be followed for OS for at least five years.

9.3 Additional evaluations

Health economics will be analyzed. In particular if the experimental arm turns out as effective as standard treatment, the savings in costs will be addressed.

10 SAFETY REPORTING

Since the study treatment consists of well-known medicinal products that are used in the approved doses and patients in the experimental arm will receive a shorter, ie less toxic regimen than the current standard treatment, no extensive safety reporting is deemed necessary. Treatment related serious adverse events (SAE) during treatment and up to 6 months after treatment will be registered. Suspected unexpected serious adverse reactions (SUSARs) will be reported by sponsor to the authorities, according to local regulations, based on the information captured in the eCRF. (E.g. SUSARs that occur in Finland shall be reported by secure e-mail (Eudralink or Fimea’s secure mail service) to FI-CTA@fimea.fi. Fimea will then transfer the information to EudraVigilance on the investigator's behalf.)

10.1 Definitions

10.1.2 Serious Adverse Event (SAE)

Serious Adverse Event (SAE): Any untoward medical occurrence in a subject to whom a medicinal product is administered that at any dose requires:

- inpatient hospitalization or prolongation of existing hospitalization,
- results in persistent or significant disability or incapacity,
- results in a congenital anomaly or birth defect,
- is life-threatening, or results in death.

Medical and scientific assessment will be made to determine if an event is serious.

10.1.3 Suspected Unexpected Serious Adverse Reaction (SUSAR)

SUSAR: An adverse reaction/event that is unexpected, serious, and suspected to be caused by the treatment, i.e. adverse reactions/events that are not included in the Reference safety information (RSI) section of the Summary of product characteristics (SmPC).

10.2.1 Assessment of causal relationship

The investigator is responsible for determining whether there is a causal relationship between the AE/SAE and use of the investigational medicinal product.

Consideration should be given to whether there is a reasonable possibility of establishing a causal relationship between the adverse event and the investigational medicinal product based on the analysis of the available evidence.

All AE can be categorized as either likely related, possibly related, unlikely related or not related, in accordance with the definitions below:

Likely related: Clinical event, including abnormal results from laboratory analyses, occurring within a reasonable time after administration of the intervention/investigational medicinal product. It is unlikely that the event can be attributed to underlying disease or other medications but is most likely caused by the investigational medicinal product and its emergence is reasonable in relationship with use of the investigational medicinal product.

Possibly related: Clinical event, including abnormal results from laboratory analyses, occurring within a reasonable time after administration of the intervention/investigational medicinal product. The event could be explained by the investigational medicinal product and its emergence is reasonable in relationship with use of the investigational medicinal product, but there is insufficient information to determine the relationship. The event could be explained by an underlying disease or other medications.

Unlikely related: Clinical event, including abnormal responses from laboratory tests, unlikely to be related to the intervention/investigational medicinal product and can be reasonably explained by other medication or underlying disease.

Not related: Clinical event, including abnormal results from laboratory analyses, that is not reasonably related to the use of the intervention/investigational medicinal product.

Those AEs which are suspected of having a causal relationship to the investigational medicinal product will be followed up until the subject has recovered or is well taken care of and on the way to good recovery.

If the reporting investigator does not provide any information on causality, the sponsor should

consult with the reporting investigator and encourage the expression of a position on this issue. The sponsor must take into account the assessment of causality provided by the investigator. If the sponsor disagrees with the investigator's assessment of causality, both the investigator's and the sponsor's views should be included in the report.

10.2.2 Reporting and registration of Serious Adverse Events (SAE)

The investigator/research nurse willshould register and document SAEs and report them to the sponsor in the eCRF. At each contact with the investigator/nurse, trial subjects should be asked about how they have been feeling since the previous visit.
The assessment that an event is an SAE ~~as well as the registration in the SAE form in the eCRF~~ must be made by the investigator.
SAEs must be reported to the sponsor in the eCRF within 24 hours of the investigator being informed of the SAE.
Follow-up information describing the outcome and handling of the SAE is reported as soon as this information is available. Any original SAE documents should be kept in the Investigator Site File.

10.2 SUSAR

Those SAE which are assessed by sponsor to be SUSARs are to be reported to the EudraVigilance database. The completed CIOMS form will be the basis for the reporting in the EudraVigilance database according to the specified time frames.
SUSAR that are fatal or life-threatening are reported as soon as possible and no later than 7 days after the SAE has become known to the sponsor. Relevant follow-up information is sent thereafter within an additional 8 days. Other SUSAR are reported as soon as possible and no later than 15 days after they have come to the sponsor’s knowledge.

Information about SUSAR occurring during the trial is compiled by the sponsor and sent to the principal investigators at all participating sites.

11 STATISTICAL METHODS

The efficacy analyses of DMFS and RFS will include all per-protocol patients (intention-to-treat population for sensitivity analysis). DMFS is defined as the time from start of treatment to detection

of distant relapse or death and RFS as the time from start of treatment to detection of relapse (local, regional or distant) or death. Kaplan-Meier techniques will be used to plot both outcomes. Comparison of outcomes between the study arms will be based on a Cox regression model. The hazard ratio (HR) associated with the study arms will be derived from this model along with the associated 95% confidence interval (CI). A Cox regression model with intervention as the exposure and the co-primary endpoints and adjusted for the tumour stage and neoadjuvant treatment (stratification variables) will be used to assess the effect. A detailed description of the Cox regression model will be specified in the Statistical Analysis Plan (SAP). The p value for testing the null hypothesis that the HR between the interventions will be greater than or equal to 1.19 will be derived from this model by comparing the log-likelihood of the fitted model with the log-likelihood of a model where the HR between the groups is set to 1.19 by use of a likelihood ratio test.

11.1 SAMPLE SIZE CALCULATIONS (CO-PRIMARY ENDPOINT – DISTANT METASTASES FREE SURVIVAL (DMFS) AND RELAPSE FREE SURVIVAL (RFS))

The study design is a non-inferiority trial investigating whether short-term adjuvant immunotherapy, is non-inferior to long-term adjuvant immunotherapy (standard of care) with a non-inferiority margin (4.0%) being accepted as being clinically appropriate in this study population.

Assumptions for the sample size calculation:

- DMFS at 2 years was 88.1% for stage IIb-c patients (Keynote 716 study) (1)
- DMFS at 2 years was 70% for stage III-IV patients (Checkmate 238 trial) (2)
- DMFS for the sample size will be based on these two measurements with a conservative estimate of 75.0% at 2 years and assuming same rates for RFS.
- Power = 80%
- Alpha level = 5%
- Non-inferiority Margin = 4%
- Accrual time = 48 months
- Follow up time = 60 months

Assuming a 4-year accrual and 5-year follow-up duration, a sample size of 1872 patients (936 in each study arm) will have 80% power (90% for each endpoint, which corresponds to a total power of 81%) with one-sided 95% confidence to declare non-inferiority with a margin of 4.0% (i.e., from 75% to 71.0% in the observation group) based on a 1:1 randomization with a corresponding HR of

1.19. The sample size may be either re-estimated or the non-inferiority boundary adjusted if the number of patients lost to follow-up is of concern.

11.2 CO-PRIMARY ENDPOINTS

Distant metastatic free survival and RFS are co-primary endpoints and to preserve the overall type I error rate, comparisons will be performed according to the following hierarchical testing:

1) The alternative non-inferiority hypothesis, H_{11}: DMFS short-term treatment is not inferior to long-term treatment (standard of care) for the first co-primary endpoint (DMFS). If H_{11} is accepted., i.e., non-inferiority is declared in (1), then the following hypothesis will be tested.
2) The alternative non-inferiority hypothesis, H12: DMFS short-term treatment is not inferior to long-term treatment (standard of care) for the second co-primary endpoint (RFS).

Each test will be performed at the 5% level of significance, with comparisons in the sequential testing procedure being conditional on the rejection of the null hypothesis of the previous comparison.

11.3 INTERIM ANALYSIS

If we allocate $\alpha = 0.005$ to the interim analysis and $\alpha = 0.045$ to the final analysis then the total sample size would increase from 1872 to 1880 subjects (940 in each arm). The calculation was made using the O'Brien-Fleming Boundary. The interim analysis will be conducted when approximately two-thirds of the total number of planned subjects are enrolled which is the optimal timing when using the O'Brien-Fleming Boundary for both co-primary endpoints (12).The results of the interim analysis will be carefully assessed by the Data safety monitoring board (DSMB) without delay and in the case of inferiority of the experimental arm the study will be prematurely terminated and all sites informed.

11.4 HANDLING OF MISSING, UNUSED, AND SPURIOUS DATA AND DEVIATIONS

Since the impact of missing data is expected to be small, no multiple imputation method for missing data is planned. However, the issue of missing data could arise; the choice of the imputation method for missing data will depend on the pattern of missing data and the type of the imputed variable. E.g. if the assumption of missing at random is true and baseline variables are missing we will use

single variable imputation, if both the independent and the outcome is missing we will consider monotonic imputation or the Markov chain Monte Carlo Method.

12 SIDE PROTOCOLS

12.2 SIDE PROTOCOL TO INVESTIGATE THE RISK OF FOOD SUPPLEMENTS

A side protocol (the MelKo study) to assess the risk of food supplements was added to the TRIM study at oncology centers in January 2021. The MelKo study ~~does not require written informed consent and~~ only comprises a questionnaire at the 6-month study visit. In April 2025~~4~~, 275~~197~~ out of the planned 420 patients had been recruited and Mel~~EL~~Ko~~O~~ will continue as a substudy to Grand SLAM.

12.3 PLASMA BIOMARKER STUDY

Schedule of sample collection

Baseline Sample	3-month Sample	6-month Sample	12-month sample	24-month Sample	36-month sample	+ sample after relapse
		mandatory thoracic and abdominal CT		mandatory thoracic and abdominal CT	~~mandatory thoracic and abdominal CT~~	
EDTA tube 6ml	EDTA tube 6ml	EDTA tube 6ml	EDTA tube 6ml	EDTA tube 6ml	EDTA tube 6ml	EDTA tube 6ml
Streck cell-free DNA tube 2x10ml	Streck cell-free DNA tube 2x10ml	Streck cell-free DNA tube 2x10ml	Streck cell-free DNA tube 2x10ml	Streck cell-free DNA tube 2x10ml	Streck cell-free DNA tube 2x10ml	Streck cell-free DNA tube 2x10ml

12.3.1 Rationale for plasma biomarker study

Cancer cells release DNA fragments into bloodstream. This cell-free ctDNA (circulating tumor DNA, ctDNA) containing tumor-specific mutations can be detected using polymerase chain reaction (PCR) or next-generation DNA sequencing (NGS). More than two-thirds of cutaneous melanomas harbor a detectable tumor mutation, usually in BRAF (35–60%) or NRAS (15–28%) genes, which can be potentially monitored by liquid biopsies (13, 14). Plasma ctDNA reflects tumor volume and metabolic activity although some tumors shed only low amounts of ctDNA (15). PCR-based methods have revealed detectable ctDNA from plasma samples in 11−37% of stage II and III melanoma patients with a known tumor mutation (16, 17). Detectable plasma ctDNA has predicted

shorter disease-free survival and overall survival in stage II and III melanomas indicating "molecular residual disease (MRD)" after complete surgical resection, and detectable ctDNA may precede radiologically detectable disease progression (16, 17).

12.3.2 Sample collection for ctDNA, proteomics, and immune cell analysis

Patients included into the Grand SLAM Study may be included into the Grand SLAM Plasma Biomarker Study after providing informed consent.

Venous blood samples will be collected in EDTA and Streck tubes and centrifuged within 30 min to extract plasma and peripheral blood white blood cells (WBC). The extracted plasma and WBCs will be immediately stored at −20°C and moved to −80°C within 2 weeks.

For droplet digital PCR (ddPCR) and next-generation sequencing (NGS) analysis, cell-free DNA will be extracted from 2 ml of plasma collected in Streck tubes using a QiaSymphony SP, following the protocol for the QiaSymphony DSP Circulating DNA Kit (Qiagen, Hilden, Germany).

For blood proteomic analyses, the extracted plasma collected in EDTA tubes will be analyzed with a proximity extension assay (Target 96 Inflammation panel, Olink Bioscience). Provided samples (0.2 ml) will be run on two separate plates in duplicate. Chemokine and cytokine profiles will be evaluated upon adjuvant treatment and follow-up. Changes in plasma protein levels will be correlated with ctDNA and disease recurrence status (disease-free, recurrence).

Peripheral blood mononuclear cells will be analyzed using flow cytometry.

12.4 OTHER SIDE PROTOCOLS

Other side protocols are allowed under prerequisite that they do not interfere with the main project. Local projects (for example tumor tissue analyses) on Grand SLAM patients may be conducted but will have to be approved by the steering group, ensuring that no interference with the main project occurs. The side-protocols have to develop their own patient information (if applicable) and ethical considerations.

13 STUDY REQUIREMENTS

13.2 STUDY ORGANIZATION

The study group consists of all principal investigators (PIs) at university as well as county hospitals. The study will be undertaken under the auspices of a steering committee. The steering group consists of the PIs at participating university hospitals. Members of the monitoring committee

(Appendix 2) will be appointed among people not directly involved in the study. In October 2023, the study protocol was presented at the meeting of the European Melanoma Group in Manchester and at the Nordic Melanoma Meeting in Reykjavik and these groups are in favor of the study.
The conduct of the study will be regularly reported and discussed at the bi-annual meetings of the Swedish Melanoma Study Group (SMSG) and at the regular meetings of the Nordic Melanoma and EORTC Melanoma Groups.
In Sweden, all seven university hospitals and the county hospitals estimated to be able to recruit at least 30 patients within three years are expected to participate. All these centers participate in the ongoing TRIM study which encompasses the same patient group as Grand SLAM. All university centers in Norway and all but one university center in Finland will join (see list above). Due to the spare of resources, a high recruitment rate can be expected. Further university hospitals in other European countries will be encouraged to participate and we expect more centers to join when the study is up and running. It is likely best that the study is organized at the oncology department but in some European countries (e.g. Germany), a dermatologist will be most suitable as PI.
Each center will appoint a PI who will be responsible for recruitment and protocol adherence. Each center is recommended to appoint a study nurse for running the study.
Satellite centers may be used in areas where it is difficult for patients to visit the study center due to distance, or when patients routinely are followed at a different clinic. The requirements for a satellite center in the study are the following:

- The center is situated within the same county (landsting/region) as the main study center.
- The PI of the main study center in that county is responsible for the satellite center and will ensure that study specific training and delegation is performed as per GCP.
- Source data will be made available by the satellite center to the main study center and registration of data in the EDC system will be completed by the main study center.
- Patient visits will be conducted at the satellite center, and assistance will be provided by the main study center when necessary.
- The satellite site has experience in immunotherapy treatment and in handling immune related adverse events.

The study will be coordinated by the clinical trial and development unit (KFUE) at Uppsala University Hospital, Sweden but since the current study will involve centers outside Sweden, more resources will be required. Hence, the project manager position at the secretariat will be increased

from 25 % to 50 % and a PhD student, 50% academic employee will be recruited. The secretariat will support the study sites, collect a log of all included patients and monitor the study. If a center does not recruit at least 50% of eligible patients, measures to increase the inclusion rate must be taken.

13.3 Costs and Funding

No industrial sponsors can be expected to volunteer in this project. Therefore, funding will be sought from private and official funds. Funds from the EU will also be considered.
The central steering group will support applications for local support.
The different countries will cover/apply for their own expenses for imaging, monitoring and study-nurses but will at the same time save a substantial amount of money since half of the patients enrolled in the study will receive systemic treatment for only 6 months instead of 12 months.
Costs for the Swedish part of the study
Salary for the project coordinator is estimated to 350 000 SEK year 1 and year 2 and 150 000 SEK for year 3, year 4 and year 5, i.e. in total 1 150 000 SEK. Costs for research nurses will likely amount to 1 050 000 year 1, year 2, year 3 and year 4 and to 800 000 SEK year 5, summing up to 5 000 000 SEK providing that 600 Swedish patients are recruited in total. A half-time clinical PhD student will cost 459 000 SEK year 1 474 000 SEK year 2, 487 000 SEK year 3, 502 000 SEK year 4 and 517 000 SEK year 5, i.e. in total 2 439 000 SEK. Monitoring costs for year 1 100 000 SEK, year 2, 3, 4 and 5 150 000 SEK, i.e. in total 700 000 SEK.
As described in section “Background”, more frequent imaging is usually performed in the adjuvant setting internationally compared to current practice in Sweden. In Great Britain the imaging scheme in Grand SLAM will not exceed current practice as the number of imaging assessments is far greater than in Sweden. One CT scan of the abdomen and thorax costs 4 000 SEK while 5 900 SEK is the cost for the baseline CT since it includes the brain. The extra Swedish scanning costs (scan at 3 years) are estimated in total to 2 400 000 SEK (600 Swedish patients). The savings in drug costs and health care resources will be much greater but since there are separate budgets for study costs and routine hospital care, all extra imaging needs to be financed within the study budget.
In addition, if the study results show that the benefit from half a year of treatment is equally effective to treatment for one year, the results will be practice changing and an enormous amount of resources will be spared world-wide.

In summary, the total cost for the Swedish part of the study amounts to 11 889 000 SEK.

14 QUALITY CONTROL AND QUALITY ASSURANCE

14.2 MONITORING

This study will be monitored by an independent study monitor before, during and after the study. The purpose of this monitoring is to ensure that this study is conducted in accordance with the protocol, and ethical and regulatory requirements and that data is collected, documented and reported as described in the current ICH-GCP version. Risk-based monitoring will be applied in this study, adapting monitoring to recruitment rate, deviations, performance etc. This will be described in detail in a separate monitoring plan. Remote monitoring will be done of all data and a limited number of on-site monitoring visits will be conducted.

14.3 SOURCE DATA

The investigator at each site is responsible for maintaining source data for each patient in the study. A document of what has been classified as source data (Source Data Location Log) in the study should be provided in the Investigator Site File, ISF. The investigator will ensure that all source documents are available for monitoring and other forms of quality control.

Source data is defined prior to study start at each study site. Source data can in some cases consist of the CRF, if specific variables are not recorded elsewhere. This will be decided in consultation with the monitor and be clearly described in the source data location log.

Monitors and auditors shall be permitted access to study-related information such as patient’s medical records, CRF and any other source data and study documents for monitoring or auditing purposes. A secrecy agreement will be signed before gaining access to medical records. The Swedish Medical Products Agency (MPA) will also be granted access in connection to inspections.

14.4 REPORTING OF DEVIATIONS AND SERIOUS BREACH

The responsible investigator shall without delay report any serious deviations (serious breach) to the sponsor of the study. Serious breach is defined as a deviation that significantly and directly affects or very likely will affect a study patient´s safety or rights, or the reliability and robustness of the data that is generated in the study. The sponsor will assess the consequence of any deviations that

have occurred and, if determined to be defined as serious breach, report to the regulatory authority via Clinical Trials Information System (CTIS) within 7 days of sponsor gaining knowledge of the breach.
Any other unforeseen events that may affect the risk/benefit balance of the study shall be reported in CTIS within 15 days of sponsor gaining knowledge of the event.

All other deviations will be summarized in the study documentation and if appropriate actions should be taken to prevet further incidences. All deviations will be reported in the clinical study report.

14.5 Adherence to study protocol, ICH-GCP and regulations

To ensure safety and integrity of included patients and quality of data, this study will be conducted in accordance with this study protocol, the Clinical Trials Regulation (536/2014), the Helsinki declaration, ICH-GCP and applicable national regulations.

15 REVIEW BY REGULATORY AND ETHICAL AUTHORITIES

The final study protocol will be approved by the Regulatory authority and Ethical review authority before this study is started. Any changes to the study protocol will be submitted to relevant authorities as applicable and in accordance with local regulatory requirements.
The investigators and institutions involved in this clinical trial confirm that they are to permit clinical trial-related monitoring, audits and regulatory inspections, including provision of direct access to source data and documents.

15.2 Informed consent

It is the responsibility of the investigator at each study site to ensure that all study patients are provided with full and adequate verbal and written information about the study, its purposes, risks and advantages as well as provisions for inclusion and exclusion. The patients must also be informed that he or she may terminate participation in the study at any time without having to provide an explanation. The patients shall be given the opportunity to ask questions and consider the information that has been provided. This information can be provided over the telephone, if required, and the patient may be given the opportunity to take the written information home to consider before accepting. If a patient chooses to accept participation in the study the informed

consent will be signed by the patient and the investigator, preferably on site however a file note can explain an alternative procedure (in the case that the approved written information sheet is sent to the patient before the site visit and the patient signs the consent form prior to the visit) on site if necessary. The patient shall receive a copy of the signed informed consent and the written information. Patients declining to participate or withdrawing will be treated according to the national guidelines i.e. the same treatment as the control group in the study.

The signed patient informed consent must be obtained before any trial related activities are performed, however this being a study that mainly follows clinical routine, information that has been gathered earlier may be retrospectively collected and used for the study.

In signing the informed consent the study patient will also consent to data being available for monitoring, audits and inspections.

If new information that may be relevant to the patients consent to participate in the study becomes available, the patient will be re-consented to participating in the study.

15.3 AMENDMENTS TO STUDY PROTOCOL

Substantial modifications/amendments to the study protocol, that may affect the wellbeing, rights of the study patient or the reliability or robustness of data, will only be adopted after an approval from ethical and/or regulatory authorities. Non-substantial amendments will be adopted upon signature of the study protocol, and included at the next substantial amendment, unless the amendment is relevant to the authorities' oversight capabilities.

16 DATA PROTECTION

The patient information will provide all patients with complete information regarding how collection, use and publication of their data will be handled in this study. The information provided in the patient information is in accordance with relevant legislation on integrity and the General data protection act and all personal information will be stored in such a manner that requirements in this legislation are adhered to. All personal data that is processed by the sponsor will be pseudonymized and identified by a study specific code.

16.2 Data collection, handling and management

In order to track all eligible patients, a system for assessing all patients diagnosed with CMM should be in place at each participating center. The reason for screening failure and poor recruitment is to be reported. Electronic CRFs for all enrolled patients are to be filled in electronically at the individual centers. All registrations (CRFs) are communicated centrally after each visit by means of an internet-based database.

The following data will be obtained on all enrolled subjects:

- Baseline demographic factors (sex and date of birth).
- Results of all diagnostic evaluations for metastatic disease during the initial assessment.
- Description of treatments; surgical, systemic adjuvant, and radiation treatment.
- Postoperative (and in case of neoadjuvant treatment, stage before start of systemic treatment) final TNM staging.
- Locoregional and distant relapses are promptly reported regardless if detected by imaging, at a follow up visit or at an unscheduled visit.

In the eCRF the patient will be coded using a study specific ID. All study patients will be registered on an enrollment log that links the name of the included study patient to the study specific ID.
All data will be registered, handled and stored in a way that enables correct reporting, interpretation and verification. An Investigator's site file with the necessary essential documents will be archived for at least 25 years. Source data in the medical records will be stored and archived in accordance with national law.

16.3 Case Report Form

An eCRF will be used for data collection in this study. It is the responsibility of the investigator to ensure that data is entered into the eCRF in a timely manner and that the data is correct and complete. A copy of the completed eCRF will be archived at the study site after the study is completed.

17 INSURANCE

Patients who participate in this study are covered by insurances according to national routines.

18 STUDY TERMINATION AND PUBLICATION

The study will be reported in CTIS no later than 15 days after termination. Within one year of the Last-patient-last visit the clinical study results will be summarized in CTIS.

19 REFERENCES

1. Eggermont AMM, Blank CU, Mandala M, Long GV, Atkinson VG, Dalle S, et al. Adjuvant pembrolizumab versus placebo in resected stage III melanoma (EORTC 1325-MG/KEYNOTE-054): distant metastasis-free survival results from a double-blind, randomised, controlled, phase 3 trial. Lancet Oncol. 2021;22(5):643-54.
2. Ascierto PA, Del Vecchio M, Mandala M, Gogas H, Arance AM, Dalle S, et al. Adjuvant nivolumab versus ipilimumab in resected stage IIIB-C and stage IV melanoma (CheckMate 238): 4-year results from a multicentre, double-blind, randomised, controlled, phase 3 trial. Lancet Oncol. 2020;21(11):1465-77.
3. Luke JJ, Rutkowski P, Queirolo P, Del Vecchio M, Mackiewicz J, Chiarion-Sileni V, et al. Pembrolizumab versus placebo as adjuvant therapy in completely resected stage IIB or IIC melanoma (KEYNOTE-716): a randomised, double-blind, phase 3 trial. Lancet. 2022;399(10336):1718-29.
4. Kirkwood JM, Del Vecchio M, Weber J, Hoeller C, Grob JJ, Mohr P, et al. Adjuvant nivolumab in resected stage IIB/C melanoma: primary results from the randomized, phase 3 CheckMate 76K trial. Nat Med. 2023;29(11):2835-43.
5. Versluis JM, Menzies AM, Sikorska K, Rozeman EA, Saw RPM, van Houdt WJ, et al. Survival update of neoadjuvant ipilimumab plus nivolumab in macroscopic stage III melanoma in the OpACIN and OpACIN-neo trials. Ann Oncol. 2023;34(4):420-30.
6. Patel SP, Othus M, Chen Y, Wright GP, Jr., Yost KJ, Hyngstrom JR, et al. Neoadjuvant-Adjuvant or Adjuvant-Only Pembrolizumab in Advanced Melanoma. N Engl J Med. 2023;388(9):813-23.
7. Blank CU, Lucas MW, Scolyer RA, van de Wiel BA, Menzies AM, Lopez-Yurda M, et al. Neoadjuvant Nivolumab and Ipilimumab in Resectable Stage III Melanoma. N Engl J Med. 2024.
8. Dummer R, Hauschild A, Santinami M, Atkinson V, Mandala M, Kirkwood JM, et al. Five-Year Analysis of Adjuvant Dabrafenib plus Trametinib in Stage III Melanoma. N Engl J Med. 2020;383(12):1139-48.
9. Iveson T, Boyd KA, Kerr RS, Robles-Zurita J, Saunders MP, Briggs AH, et al. 3-month versus 6-month adjuvant chemotherapy for patients with high-risk stage II and III colorectal cancer: 3-year follow-up of the SCOT non-inferiority RCT. Health Technol Assess. 2019;23(64):1-88.
10. Earl H, Hiller L, Vallier AL, Loi S, McAdam K, Hughes-Davies L, et al. Six versus 12 months' adjuvant trastuzumab in patients with HER2-positive early breast cancer: the PERSEPHONE non-inferiority RCT. Health Technol Assess. 2020;24(40):1-190.
11. Helgadottir H, Ny L, Ullenhag GJ, Falkenius J, Mikiver R, Bagge RO, et al. Survival after Introduction of Adjuvant Treatment in Stage III Melanoma: A Nationwide Registry-Based study. J Natl Cancer Inst. 2023.
12. Togo K, Iwasaki M. Optimal timing for interim analyses in clinical trials. J Biopharm Stat. 2013;23(5):1067-80.
13. Cancer Genome Atlas N. Genomic Classification of Cutaneous Melanoma. Cell. 2015;161(7):1681-96.
14. Lee JH, Choi JW, Kim YS. Frequencies of BRAF and NRAS mutations are different in histological types and sites of origin of cutaneous melanoma: a meta-analysis. Br J Dermatol. 2011;164(4):776-84.

15. Keller L, Belloum Y, Wikman H, Pantel K. Clinical relevance of blood-based ctDNA analysis: mutation detection and beyond. Br J Cancer. 2021;124(2):345-58.

16. Lee RJ, Gremel G, Marshall A, Myers KA, Fisher N, Dunn JA, et al. Circulating tumor DNA predicts survival in patients with resected high-risk stage II/III melanoma. Ann Oncol. 2018;29(2):490-6.

17. Tan L, Sandhu S, Lee RJ, Li J, Callahan J, Ftouni S, et al. Prediction and monitoring of relapse in stage III melanoma using circulating tumor DNA. Ann Oncol. 2019;30(5):804-14.

17.Malmberg R, Zietse M, Dumolin DW, Hendrix JJMA, Aerts JGJV, van der Veldt AAM, Koch BCP, Sleijfer S, van Leeuwen RWF. Alternative dosing strategies for immune checkpoint inhibitors to improve cost-effectiveness: a special focus on nivolumab and pembrolizumab. Lancet Oncol 2022; 23: e552-61

20 APPENDICES

1. Schedule of assessments
2. Data and Safety Monitoring Board (DSMB) charter

APPENDIX 1: Schedule of assessments – These are study specific assessments, and all routine follow up assessments according to national guidelines must also be performed.

**Assessments:**	**Baseline (within 28 days of study treatment start)**	**3 months +/- 28 days (g) Only biomarker patients**	**6 months +/- 28 days (g)**	**12 months +/- 28 days (g)**	**18 months +/- 28 days (g)**	**24 months +/- 28 days (g)**	**30 months +/- 28 days (g)**	**36 months +/- 28 days (g)**	**48 months +/- 28 days (g)**	**60 months +/- 28 days (g)**
	Adjuvant treatment is started within 12 weeks after last melanoma related surgery (d)									
Informed consent (a)	X									
Inclusion/exclusion criteria	X									
Demographics	X									
Cancer/treatment history (b)	X									
Concomitant medication	X		X	X	X					
Physical examination (c)	X		X	X	X	X	X	X		
CT scan thorax-abdomen-brain/MRI brain or i.v. contrast enhanced whole body FDG-PET-CT including brain or whole body FDG-PET-CT + MRI brain (e)	X (f)		X			X				
Overall survival follow-up									X	X
SAE		X (h)	X (h)	X (h)	X (h)					
Optional sub-study MelELKoO			X							
Optional biomarker substudy (i)	X	X	X	X		X	X			
Pregnancy test[j]	X	X	X[k]	X[k]						

a) Written informed consent must be obtained before any study-specific screening procedures are performed.
b) Includes thickness of primary tumour, number of examined nodes/nodes with metastases, TNM staging, postoperative treatment (i.e. adjuvant radiotherapy).
c) To be performed according to clinical routines and this is the follow-up schedule according to current Swedish guidelines for stage III disease.
d) Final surgery is defined as excision and/or sentinel node biopsy, lymph-node dissection or metastasectomy. For neoadjuvant treated patients, randomization is performed within 6 weeks.
e) Brain must be included in the baseline scan and at 24 months. To be performed according to clinical routines and this is the mandatory minimum schedule.

f) To be performed within 28 days for adjuvant and 8 weeks for neoadjuvant patients respectively prior to study treatment startrandomization.
g) Time points from study treatment start, +/- 8 weeks allowed for neoadjuvant treated patients.
h) Treatment related serious adverse events during treatment and up to 6 months after treatment will be reported.
i) And at relapse.
j) Highly sensitive urine or serum/plasma pregnancy test for WOCBP.
k) Only applicable for standard arm.

APPENDIX 2:

Data and Safety Monitoring Board (DSMB) Charter

<u>DSMB organization:</u>

Principal Investigator	Prof. Gustav Ullenhag<br>Uppsala University Hospital<br>Gustav.ullenhag@igp.uu.se
Site-specific Principal Investigator	
Sponsor Representative	Gustav Ullenhag
Project Managers	Leila Boukharta
Biostatistician	Anders Berglund

Independent Members (subjected to change during the trial):
Prof. Gunilla Enblad, Prof. Simon Ekman, Prof. Peter Nygren

Background and purpose of this plan

A prospective randomized international multicenter study to compare short versus long adjuvant immunotherapy after radical surgery of stage IIb-c, III and IV cutaneous malignant Melanoma. The study is carried out in Sweden, Norway, Finland and potentially other countries will be added later on.

In order to ensure patient safety, this communication plan has been developed to describe the flow of information between the involved parties (Sponsor, Principal Investigator, and in-dependent DSMB members).

DSMB review

The DSMB will provide recommendations about stopping or continuing the trial based on the criteria that have been defined in the study protocol (in the case of inferiority of the experimental arm in the interim analysis or for any safety reasons affecting the risk-benefit balance). The following information should be provided to the DSMB one (1) week prior to the DSMB-meeting.

1. Study conduct
    a. protocol adherence (reporter protocol deviations)
    b. patient withdrawals
    c. distribution between treatment groups
2. Safety monitoring
    a. SAE:s and SUSAR:s
    b. New information that has been published if applicable
    c. The results of the interim analysis (done when approximately two-thirds of the total number of planned subjects are enrolled)

However, the DSMB may for any concerns regarding safety recommend stopping the trial.

It is the responsibility of the Sponsor, based on the advice provided by the DSMB, to decide whether a premature end of the study will be made.

Schedule

DSMB members will be called for meetings in the following cases:

- Close to FPI for an initial meeting
- Once a year while the study is ongoing.
- Immediately after finalisation of the interim analysis.
- At any time when safety issue occurs and upon the Principal Investigator’s demand.

Attendance

At least one of the independent members should attend the official DSMB meeting.

Meeting minutes

Minutes of all official meetings of the DSMB will be part of the Sponsor’s trial master file.

Confidentiality

All information discussed during DSMB meetings should be treated as confidential.

Meeting preparation and plan

- Investigator(s) is responsible to collect all relevant information and safety data, and submit the data to Sponsor.
- The Sponsor or project manager distributes the prepared material (as described under DSMB review) to DSMB members 1 (one) week prior to the meeting, and calls for a meeting.
- DSMB members meet on-line, by telephone or in person and discuss the data, meeting minutes will be written and filed.
- A recommendation to continue or stop the trial will be made during or after the meeting by the independent DSMB members.

Other related actions

If an SAE is classified as a SUSAR (Suspected Unexpected Adverse Reaction), the sponsor will report it to the authorities (within 7 days if life-threatening or death, or within 15 days if not life-threatening).

- Depending on the nature of the safety information, and if, in the opinion of the Investigator, the event may contribute to the safety evaluation for future treatment, the Investigator and Sponsor may at any time have an on-line, telephone or face-to-face meeting with the DSMB for advice regarding safety issues. Relevant parties (e.g., Project Manager) are invited to discuss safety issues to ensure that essential safety information is shared within the study team. The meetings will be documented and archived in the Trial Master File.
- Once a year a Development Safety Update Report (DSUR) will be compiled by the Sponsor with the help of the Project Manager at KFUE and submitted to the regulatory authorities.
