# Supplementary material for: Grand SLAM study protocol: a prospective randomised multicentre study of shortened versus standard duration adjuvant immune checkpoint inhibition for stage IIB-C, III and IV cutaneous melanoma
Source: BMC Cancer. 2026 Mar 27;26:460. doi: 10.1186/s12885-026-15924-4 (PMC13064366; doi:10.1186/s12885-026-15924-4)
Supplement: Supplementary file 3 — Supplementary Material 3. [file 12885_2026_15924_MOESM3_ESM.pdf]

**Supplementary file 1:** Centres that are recruiting patients by 2026-03-16

Uppsala

Stockholm

Skåne

Gothenburg

Örebro

Linköping

Västerås

Karlstad

Eskilstuna

Gävle

Falun

Sundsvall

Jönköping

Kalmar

Oslo

Haukeland

Trondheim

Stavanger

Ålesund

Kristiansand

Helsinki

Turku
